# Identification of meibomian gland testosterone metabolites produced by tissue-intrinsic intracrine deactivation activity

**DOI:** 10.1016/j.isci.2025.111808

**Published:** 2025-01-27

**Authors:** Khanh Tien Nguyen Pham, Takahito Miyake, Tomo Suzuki, Shigeru Kinoshita, Yuki Hamada, Hikari Uehara, Mamiko Machida, Takeshi Nakajima, Emi Hasegawa, Masao Doi

**Affiliations:** 1 Department of Systems Biology, Graduate School of Pharmaceutical Sciences, Kyoto University, Sakyō-ku, Kyoto 606-8501, Japan; 2 Department of Ophthalmology, Kyoto Prefectural University of Medicine, Kamigyō-ku, Kyoto 602-0841, Japan; 3 Department of Ophthalmology, Kyoto City Hospital, Nakagyō-ku, Kyoto 604-8845, Japan; 4 Department of Frontier Medical Science and Technology for Ophthalmology, Kyoto Prefectural University of Medicine, Kamigyō-ku, Kyoto 602-0841, Japan; 5 Senju Laboratory of Ocular Sciences, Senju Pharmaceutical Co., Ltd., Kobe 650-0047, Japan

**Keywords:** Biochemistry, Molecular Biology, Physiology

## Abstract

Intracrinology—wherein hormones are synthesized in the organ where they exert their effect without release into circulation—has been described. However, molecular mechanisms of hormone deactivation within intracrine tissue are still largely unknown. The meibomian glands in the eyelids produce oil (meibum) to the ocular surface to prevent dehydration (dry eye). Androgens are generated inside this gland and are crucial for its tissue-homeostasis. However, there is no data showing the presence of androgens in meibum, implying local conversion/deactivation into unknown metabolites. Here, we performed radioactive tracer studies in combination with pharmacological enzyme inhibition, followed by targeted liquid chromatography-tandem mass spectrometry (LC-MS/MS) analysis, and found three androgen metabolites—androstanedione, androsterone, and epiandrosterone—in mouse and human meibomian glands. Accounting for the enzymatic conversion, we show tissue-endogenous 3α/3β-ketosteroid reductase expression. We therefore reinforce the idea that androgens are metabolically inactivated within the glands. These metabolite markers may help to assess meibomian local androgen activity using meibum.

## Highlights

• Eyelid meibomian glands endogenously deactivate testosterone
• The meibomian gland testosterone metabolites are conserved between humans and mice
• There is no sex-related difference in meibomian testosterone metabolism
• Enzymes for testosterone production and deactivation are present in meibomian cells

## Graphical abstract

**Figure:**
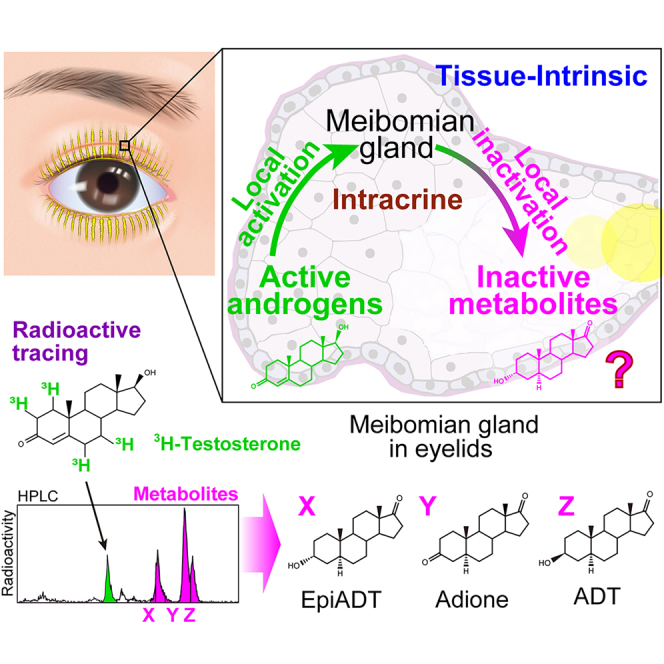

## Introduction

Canonically, hormones are produced by endocrine organs and delivered to distal target tissues. However, for steroids, the concept of “intracrinology,” whereby hormones are synthesized in the tissue where they exert their effect without release into circulation, has been described^1^^,^^2^^,^^3^ for a number of extra-gonadal tissues such as uterine endometrium^4^^,^^5^^,^^6^ and skin sebaceous gland^7^^,^^8^ as well as several cancerous tissues including those of the prostate,^9^^,^^10^ breast,^11^^,^^12^ and bone.^13^ Yet, the mechanisms by which active steroid hormones are degraded in intracrine tissues are still not fully understood. The degradation or deactivation of bioactive steroids is as crucial as the synthesis of new ones in determining the timing and location of their biological effects *in vivo*. Our study focuses on the degradation process of steroid hormones in the meibomian gland, a recently recognized intracrine tissue^14^ situated in the tarsal plate of the eyelids.

The meibomian glands are responsible for producing and secreting an oily substance called meibum. This meibum oil forms a crucial part of the tear film that covers the ocular surface. The primary function of meibum is to slow down the evaporation of the tear film, thereby preventing the eyes from drying out and maintaining proper lubrication for optimal vision. Therefore, dysfunction of the meibomian glands leads to dry eye conditions and other ocular surface disorders; particularly, it is recognized that meibomian gland dysfunction is clinically the most common cause of evaporative dry eye disease.^15^^,^^16^^,^^17^^,^^18^^,^^19^^,^^20^^,^^21^

Androgens are generated or activated inside the meibomian glands in both females and males, and these hormones are crucial to promote meibum production.^15^ The meibomian gland acinar cells express the androgen receptor (AR)^22^^,^^23^ and its activation causes upregulation of meibum production through a holocrine mechanism.^14^^,^^15^ Androgen deficiency—such as that caused by antiandrogen clinical treatment—has been reported to be associated with the development of meibomian gland dysfunction.^24^^,^^25^ Thus, evidence suggests that measuring androgen activity within the meibomian gland could be clinically relevant in understanding the etiology of meibomian gland dysfunction and its related dry eye symptoms.^26^^,^^27^ However, at present, there is no clinical biomarker to reflect local androgen activity in the meibomian gland.

At the molecular level, we previously demonstrated that the meibomian gland acinar cells express the type I 3β-hydroxysteroid dehydrogenase (type I 3β-HSD, or HSD3B1), which allows on-site production of androgens (conversion of their precursor substrates to active androgens) in the meibomian gland.^14^ These locally activated hormones are believed to function only in the meibomian gland, because they are likely inactivated in the same tissue. This localized hormone action likely ensures that hormones exert their effects only in the tissue where they are produced. While part of the mechanism of androgen synthesis in the meibomian gland has been elucidated,^14^^,^^28^ the equally important process of hormonal inactivation remains less understood. Specifically, the molecular identity of androgen metabolites and the enzymes responsible for their inactivation are still unknown.

There are comprehensive lipidome studies characterizing the meibum in humans; however, none reported the existence of androgens, such as testosterone (Te), in human meibum samples tested.^29^^,^^30^^,^^31^^,^^32^^,^^33^^,^^34^^,^^35^ This absence may be due to the low levels of Te, possibly because it is inactivated into other metabolites. The current study, therefore, aimed to identify tissue-endogenous Te metabolites in human and mouse meibomian gland. To this end, we first traced candidate Te metabolites in mice and then applied these findings to humans by analyzing human meibum metabolites.

## Results

### Tracing testosterone metabolites

**Figure 1:**
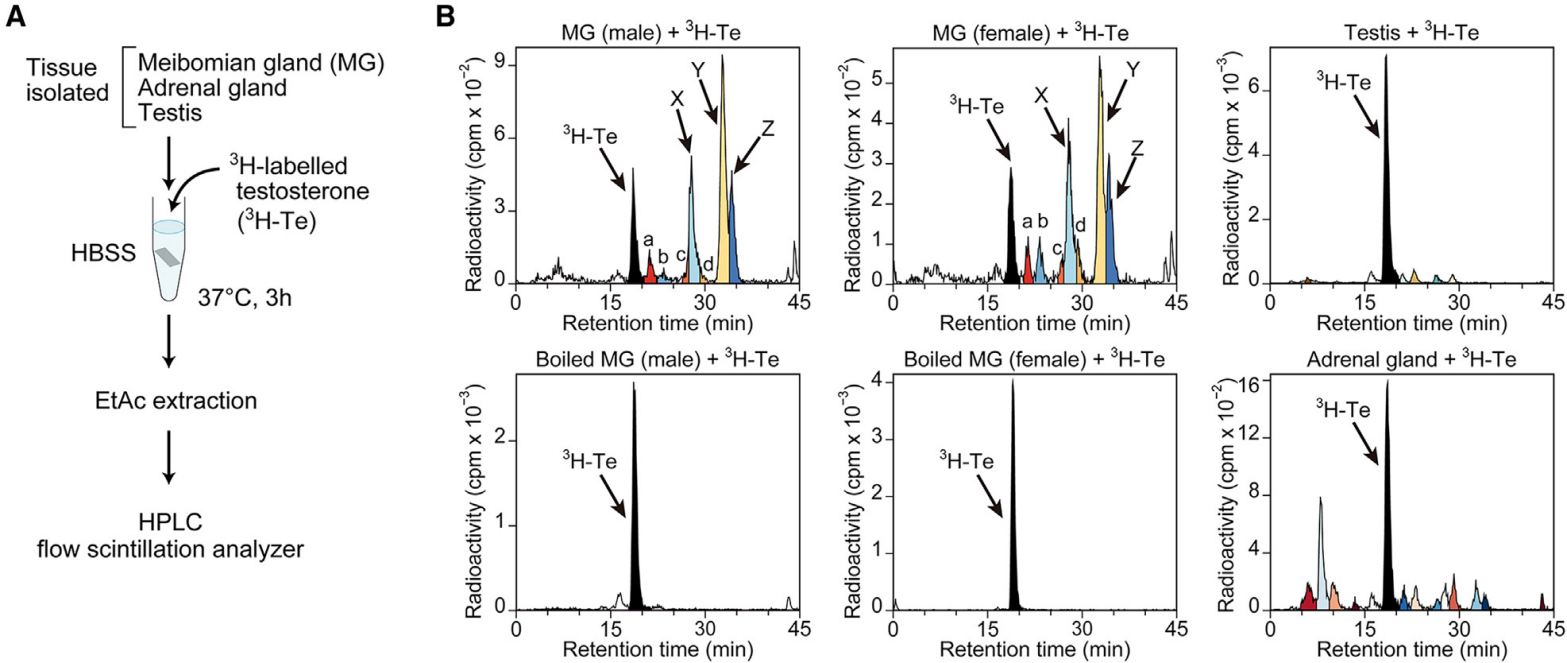
Tracing of tissue-endogenous testosterone metabolism in the meibomian gland (A) Schematic illustration of radioisotopic testosterone tracing. Freshly isolated mouse tissues were incubated *in vitro* with ^3^H-labelled testosterone (^3^H-Te) and its metabolites were detected using an HPLC-flow scintillation analyzer. (B) Representative chromatograms showing metabolites of testosterone in the meibomian gland of male and female mice. Boiled male and female meibomian gland tissues serve as control. Metabolites in the adrenal gland and testis were analyzed in comparison. Peaks labeled as X, Y, Z, a, b, c, and d in the meibomian gland were analyzed in Figures 2 and 3.

To gain insight into previously uncharacterized tissue-endogenous metabolism or catabolism of testosterone within the meibomian gland, we initiated our study by performing radioisotopic tracer experiments using mouse tissues incubated with radioactive tritium-labeled testosterone (^3^H-Te) (Figure 1A). Metabolites were separated by HPLC, followed by radioisotope measurement using an online-linked flow scintillation analyzer (see STAR Methods). Freshly isolated mouse whole meibomian gland tissues, incubated *in vitro* with ^3^H-Te, produced three dominant radioactive metabolites with retention times of 28 min (peak X), 33 min (peak Y), and 34 min (peak Z), with minor or trace amounts of radioactive products around 21 min (peak a), 23 min (b), 27 min (c), and 29 min (d), all of which were undetectable in boiled meibomian tissue (see Figure 1B). There was no obvious sex-related difference as revealed by similar radioactive products of ^3^H-Te in the meibomian gland of female mice. In comparison, testosterone (^3^H-Te) largely remained unconverted after incubation with the isolated testis or the adrenal gland. A different product peaking around 8 min was mainly observed in the adrenal gland (Figure 1B), indicating that the meibomian gland possesses a tissue-unique and potent metabolic activity against testosterone.

### Effects of blocking 5α-reductase and aromatase on meibomian gland Te metabolites

**Figure 2:**
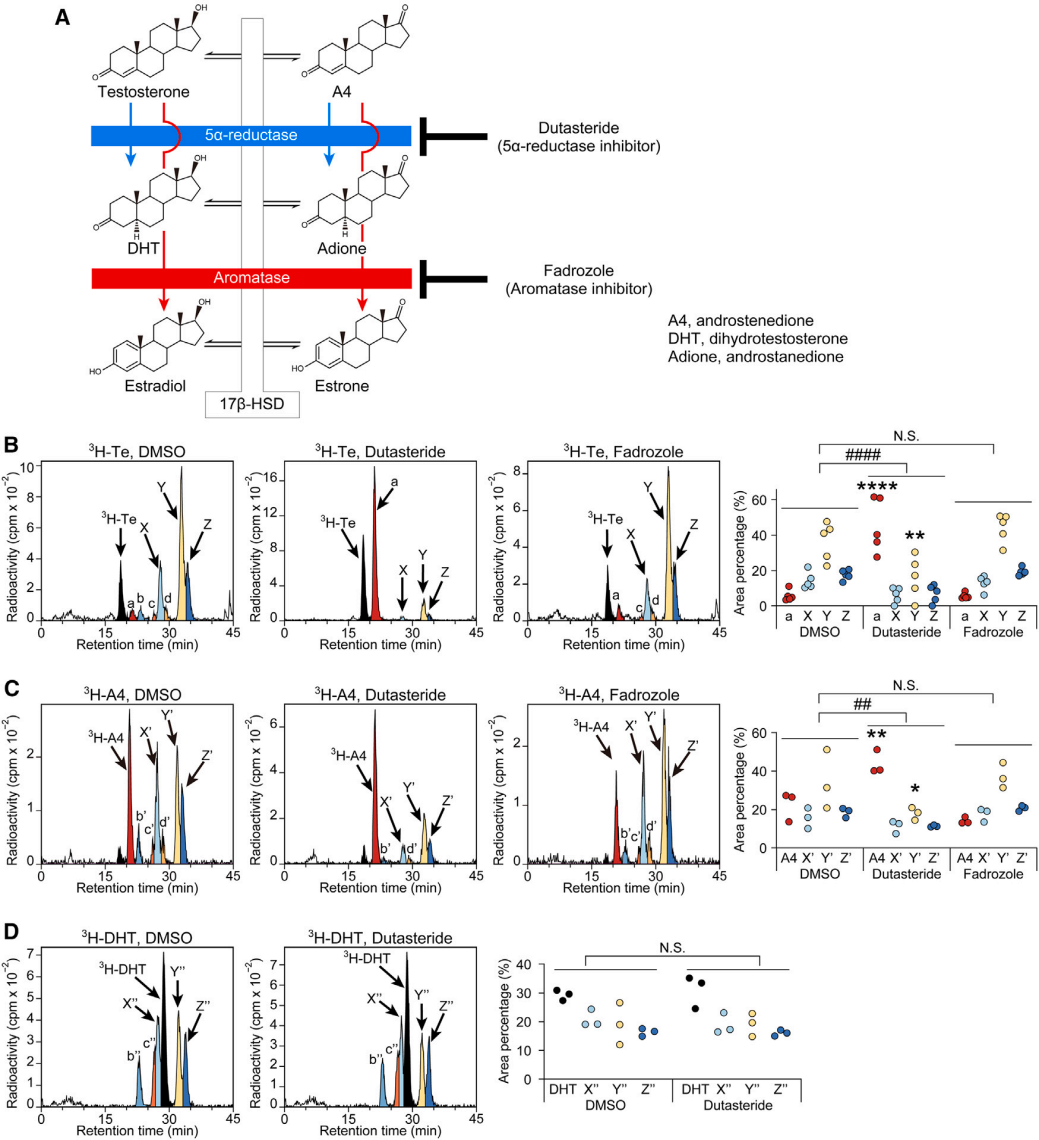
Effects of pharmacological inhibition of 5α-reductase by dutasteride and aromatase by fadrozole on the testosterone metabolism in the meibomian gland (A) Potential metabolic pathway of Te and A4 via 5α-reductase (blue) and aromatase (red). (B) Example chromatograms and peak-area ratio of ^3^H-Te metabolites in the meibomian gland incubated with dutasteride, fadrozole or vehicle (0.5% DMSO) (*n* = 5 for each condition). (C) Example chromatograms and peak-area ratio of ^3^H-A4 metabolites in the meibomian gland incubated with dutasteride, fadrozole or vehicle (0.5% DMSO) (*n* = 3 for each condition). (D) Example chromatograms and peak-area ratio of ^3^H-DHT metabolites in the meibomian gland treated with dutasteride or vehicle (0.5% DMSO) (*n* = 3 for each). Data in (B), (C) and (D) were analyzed using two-way analysis of variance (ANOVA) followed by Bonferroni’s post hoc test. ANOVA interaction: ##*p* < 0.01, ####*p* < 0.0001, N.S., not significant. ∗*p* < 0.05, ∗∗*p* < 0.01, ∗∗∗∗*p* < 0.0001 versus each DMSO control. A4, androstenedione; DHT, 5α-dihydrotestosterone.

To gain insights into the nature of meibomian gland Te metabolites, we first examined the influence of blocking 5α-reductase and aromatase on the appearance of each product (Figure 2A). We observed that all products except a (that is, X, Y, Z, b, c, and d) were profoundly reduced by the treatment of dutasteride, a 5α-reductase inhibitor (Figure 2B). The peak area ratios of X, Y, and Z were significantly decreased upon dutasteride treatment relative to those of vehicle control, with an accompanied significant increase of the peak area of a (Figure 2B). On the other hand, fadrozole, a specific inhibitor for aromatase, did not produce statistically significant effect on the appearance of metabolites (Figure 2B), indicating that the metabolites X, Y, Z, and others were raised through 5α-reductase, not aromatase, and that the metabolite a was produced at the expense of the inhibited 5α-reductase-mediated metabolism to X−Z.

### Androstenedione and 5α-dihydrotestosterone are not metabolites X, Y, and Z

Next, the RI tracer experiments were performed using different substrates (Figures 2C and 2D). Androstenedione (A4) is a potential Te metabolite that could accumulate upon the blockade of 5α-reductase (see Figure 2A). We thus assumed that the product a might be A4. Consistently, ^3^H-labeled A4 (^3^H-A4) not only had the same retention time as the metabolite a in the chromatogram but also produced three dominant metabolites, most likely X, Y, and Z, in a manner that depended on 5α-reductase activity (Figure 2C).

We also speculated that 5α-dihydrotestosterone (DHT) might be included among the major metabolites X−Z. However, this was not the case. ^3^H-labeled DHT eluted at a similar elution time as d and was converted into radioactive products resembling X, Y, Z, b, and c (but not a) (Figure 2D); These conversions were not affected by dutasteride treatment (Figure 2D), consistent with DHT being already a 5α-reduced product of Te (see a metabolic pathway, Figure 2A). These data suggest that the three major metabolites, X, Y, and Z, are likely 5α-reduced testosterone metabolites or their derivatives, distinct from DHT.

### Mass spectrometric identification of meibomian gland Te metabolites

**Figure 3:**
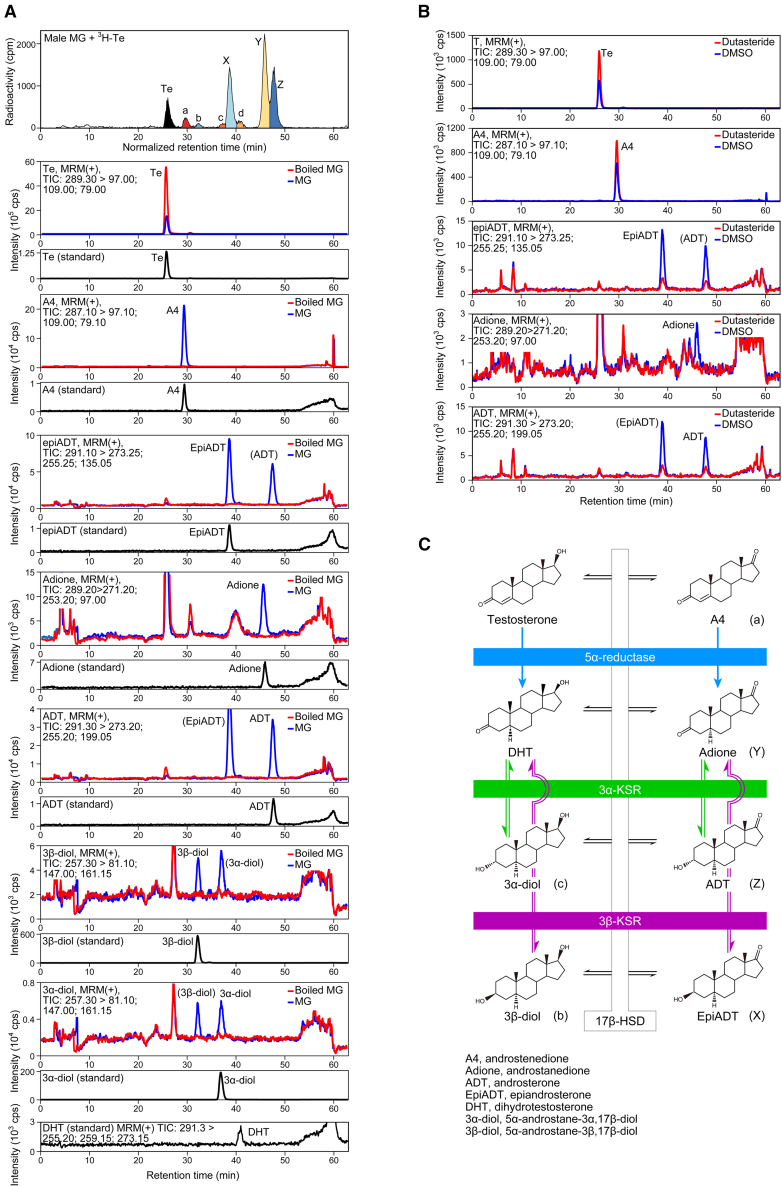
Mass spectrometric characterization of testosterone metabolites produced by the meibomian gland (A) LC-MS/MS MRM total ion chromatograms of testosterone (Te) and potential testosterone metabolites, androstenedione (A4), epiandrosterone (epiADT), androstanedione (adione), androsterone (ADT), 5α-androstane-3β,17β-diol (3β-diol) and 5α-androstane-3α,17β-diol (3α-diol), of testosterone (non-radioactive)-treated meibomian gland (blue) and those with boiled meibomian gland (red). For comparison, RI chromatography was performed in parallel using the same LC method (top). Authentic standards serve to verify the retention time of metabolites (black). b, 3β-diol; c, 3α-diol; d, not determined. (B) LC-MS/MS chromatograms of Te, A4, epiADT, adione and ADT of testosterone-treated meibomian gland with or without dutasteride application. (C) Proposed testosterone metabolic pathway in the meibomian gland.

To characterize the identity of meibomian gland Te metabolites, we next utilized targeted liquid chromatography-tandem mass spectrometry (LC-MS/MS) approach (Figure 3). To this end, we searched for the presence of androstanedione (adione), androsterone (ADT), epiandrosterone (epiADT), DHT, 5α-androstane-3α,17β-diol (3α-diol), and 5α-androstane-3β,17β-diol (3β-diol), all of which are potential 5α-reduced Te metabolites, in addition to A4—the 17-oxidized metabolite (metabolite a) tested in Figure 2C. Non-radioactive Te was incubated *in vitro* with isolated meibomian tissues, and metabolites were identified by comparing the mass spectra and retention times with those of the authentic standards (Figures S1 and 3A). In the uppermost chromatogram in Figure 3A, radioactive ^3^H-Te metabolites were separated using the same LC conditions as the mass spectrometry to facilitate comparison of the metabolites’ retention times. With these conditions, we obtained LC-MS/MS chromatograms consistent with the idea of the Te metabolites X, Y, Z, a, b, and c being epiADT, adione, ADT, A4, 3β-diol, and 3α-diol, respectively (Figure 3A). All these metabolites were not produced in boiled meibomian gland (Figure 3A, *red* chromatograms). Moreover, in dutasteride-treated meibomian glands (Figure 3B), the production of epiADT (X), adione (Y), and ADT (Z) was suppressed, while A4 (a) and Te accumulated, compatible with the data in Figure 2B, which supports the identification of X, Y, and Z as epiADT, adione, and ADT, respectively. Our data therefore suggest that in the meibomian gland, the 5α-reduced A4 metabolite, which is adione (Y, see a schematic in Figure 3C), is converted into two distinct enantiomeric metabolites, ADT (Z) or epiADT (X), through the enzymatic reaction via the 3α-ketosteroid reductase (3α-KSR) or 3β-ketosteroid reductase (3β-KSR), respectively (Figure 3C).

### Detection of Te metabolites in human meibum

**Figure 4:**
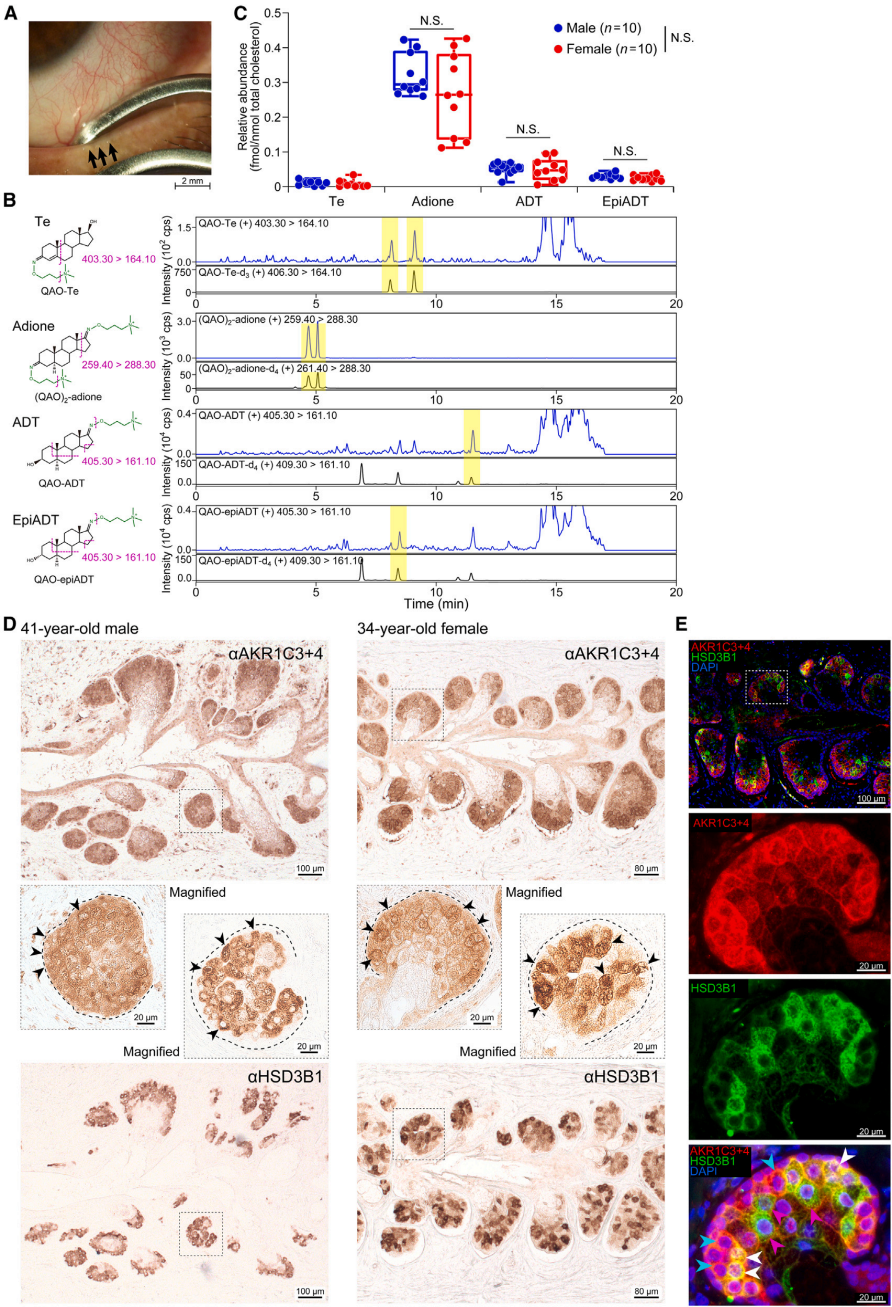
Human meibum testosterone metabolites and corresponding enzyme expression in the meibomian gland (A) Meibum collection. (B) Representative LC-MS/MS chromatograms showing QAO-derivatized Te, adione, ADT, and epiADT in human meibum specimen with chemical structure of each QAO-derivative shown on the left. (C) Relative concentration of Te, adione, ADT, and epiADT in meibum specimen from men and women. For the boxplots, the center line indicates medians, the box boundaries mark the 25^th^ and 75^th^ percentiles, and the whiskers indicate minima and maxima. *n* = 10 per gender. (D and E) 3α-/3β-KSR expression in human meibomian gland. (D) Anti-AKR1C3+AKR1C4 (for human 3β-KSR and 3α-KSR) and anti-HSD3B1 immunohistochemistry using a pair of flip-flopped mirror image serial section from 41-year-old male and 34-year-old female eyelid specimen. The dotted boxes indicate the region of magnified view. Representative AKR1C3/AKR1C4-immunopositive cells and HSD3B1-immunopositive cells are pointed by arrowheads. (E) Double-label immunofluorescence of AKR1C3+4 (red) and HSD3B1 (green). The dotted box, the region of magnified view. White arrowheads, AKR1C3+4- and HSD3B1-double positive cells. Blue arrowheads, AKR1C3+4-positive and HSD3B1-negative cells. Pink arrow heads, HSD3B1-dominant cells. Specimen, 34-year-old female meibomian gland. Data in (C) was analyzed using two-way ANOVA followed by Bonferroni’s post hoc test. N.S., not significant.

Based on our initial motivation, we finally inquired whether the identified three major Te metabolites can be found in meibum samples taken from human eyelids (Figure 4). Meibum samples were obtained from healthy volunteers as described in STAR Methods (see also Figure 4A). To enhance the MS/MS sensitivity, we employed a quaternary aminooxy (QAO) reagent to derivatize the carbonyl functional group of Te, epiADT, adione, and ADT (see Figure 4B, *left*). Te was undetectable or below the lower limit of quantitation (LOQ) in almost all human meibum samples tested (Figure 4C; see also Table S1). On the other hand, adione, ADT, and epiADT (the three major mouse Te metabolites) were all readily detectable with an average concentration normalized by total cholesterol of 0.30 ± 0.02 (SEM) fmol/nmol for adione, 0.05 ± 0.005 fmol/nmol for ADT, and 0.03 ± 0.002 fmol/nmol for epiADT. There was no sex-related difference in the amount of metabolites (mean ± SEM, fmol/nmol, for adione, male, 0.32 ± 0.02, female, 0.27 ± 0.04; ADT, male, 0.05 ± 0.005, female, 0.05 ± 0.01; epiADT, male, 0.03 ± 0.003, and female, 0.02 ± 0.003) (Figure 4C), reminiscent of the similarity between male and female Te metabolite profiles traced in mice (Figure 1B).

As shown in Figure 4D, existence of ADT and epiADT in human meibum samples was further corroborated by immunostaining of the corresponding enzyme; We used a specific antibody to AKR1C3 and AKR1C4 (anti-AKR1C3/4) because the enzymes encoded by human *AKR1C3* and *AKR1C4* are reported to exhibit 3α/3β-KSR activity *in vitro*^36^^,^^37^ and expression surveys using accessible transcriptome datasets for human meibomian gland tissue and cells point to the presence of these enzymes among the other AKR1C subtypes at the mRNA level.^38^^,^^39^ We also used a specific antibody to HSD3B1, which is the enzyme essential for producing androgen^40^ and has been demonstrated to be present in the meibomian gland cells of both men and women,^14^ for comparison.

Immunohistochemistry (Figure 4D) revealed strong anti-AKR1C3/4 immunoreactivities within the meibomian gland acinar cells, but not in duct/ductule cells or adjacent connective tissues. Anatomically, each single meibomian gland is composed of multiple acini connected via short ductules to a long central duct that extends to the orifice at the eyelid margin^17^^,^^41^: anti-AKR1C3/4 immunoreactivities were detected in nearly all acini and broadly distributed within each of them (Figure 4D). A similar staining pattern was also observed using a different antibody (see Figure S2). Moreover, no obvious difference was observed between male (Figure 4D, *left*) and female (Figure 4D, *right*) eyelids, consistent with the detection of similar metabolites in both sexes.

The expression of HSD3B1, an androgen-producing enzyme, was distributed in acini but in a mosaic distribution pattern,^14^ resulting in the observation that nearly all HSD3B1-immuno-positive cells (*green*) are also immunopositive for AKR1C3/4 (*red*), but not vice versa (Figure 4E, dual-label immunofluorescence for HSD3B1 and AKR1C3/4) (see also serial flip-flop tissue sections for HSD3B1 and AKR1C3/4, Figure 4D). It is likely that Te in the meibomian gland is metabolized inside as well as outside of the HSD3B1-positive cells by AKR1C3/4.

## Discussion

The current study was conducted to approach the identity of previously uncharacterized Te metabolite-species contained in the human meibomian gland’s secretion. Not surprisingly, Te itself was not detected at a quantifiable concentration in human meibum samples tested.^29^^,^^30^^,^^31^^,^^32^^,^^33^^,^^34^^,^^35^ To approach the unknown metabolites, we took advantage of the hypothetical resemblance of mouse and human meibomian gland and traced the tissue-endogenous Te metabolism using the mouse meibomian gland tissue, which eventually led to identifying three Te metabolites, adione (androstanedione), epiADT (epiandrosterone), and ADT (androsterone) in human and mouse meibomian gland. No obvious sex-related difference was observed. These results support the notion that humans and mice exhibit similar tissue-endogenous androgen inactivation profiles in the meibomian gland (see Figure S3, a schematic summary of our findings).

Intracrine activity does not solely refer to the activity of hormone production *in situ* but it also denotes the activity of hormone inactivation inside the tissue where the hormone is produced and acts.^1^^,^^2^^,^^3^^,^^4^^,^^5^^,^^6^^,^^7^^,^^8^^,^^9^^,^^10^^,^^11^^,^^12^^,^^13^^,^^42^^,^^43^^,^^44^^,^^45^^,^^46^ Previously, we elaborated on the aspect of hormone production by showing the presence of 3β-HSD enzyme responsible for the local androgen production in the meibomian gland^14^; however, the mechanism of hormone inactivation limb remains poorly understood. In the present study, we identified detectable amounts of three specific Te metabolites—epiADT, ADT, and adione—in human meibum samples. We also found that human meibomian gland acinar cells strongly express 3α/3β-KSR (AKR1C3/4), which aligns with the detection of epiADT and ADT. This enzyme catalyzes 3-ketosteroid reduction to inactivate androgens.^37^ Of interest, the expression of 3α/3β-KSR (AKR1C3/4) was more broadly distributed than that of 3β-HSD. This spatial relationship between 3β-HSD (androgen-activating enzyme) and 3α/3β-KSR (androgen-inactivating enzyme) supports the concept of robust testosterone metabolism involving both 3β-HSD-positive and negative cells (Figure S3). The large inactivation area surrounding the activation site likely explains the loss of detectable levels of Te during meibum secretion. In addition, the time that this secretion (holocrine) takes reaches as long as ∼9 days, as reported in rats,^47^ providing an additional reason for undetectable levels of Te in the meibum. This enzymatically robust deactivation system may also apply to other tissues; in skins, 3α/3β- KSR is reported to broadly distribute in epidermis,^48^ while 3β-HSD is confined to the sebaceous gland.^49^ In breast and prostate cancers, the expression of deactivation enzymes (such as estrogen sulfotransferase and glucuronosyltransferase) is negatively correlated with tumor progression and malignancy.^50^^,^^51^ These findings align with the principle of intracrinology, wherein hormones are inactivated within the same tissue where they are synthesized, thereby limiting the influence of bioactive hormones to the local region in which they are produced,^1^^,^^2^^,^^3^^,^^4^^,^^5^^,^^6^^,^^7^^,^^8^^,^^9^^,^^10^^,^^11^^,^^12^^,^^13^^,^^42^^,^^43^^,^^44^^,^^45^^,^^46^ and potential dysregulation of this system may lead to pathological conditions.

A more precise explanation may be necessary for clarifying why the meibomian Te metabolite species remained unidentified till our study. Indeed, despite comprehensive lipidome studies conducted for human meibomian gland excreta (meibum), adione, ADT, epiADT have so far escaped identification.^29^^,^^30^^,^^31^^,^^32^^,^^33^^,^^34^^,^^35^ This is likely largely due to the scant presence of Te metabolites, which are the catabolites of biological hormones, in the meibum, compared to other lipid constituents: cholesteryl esters (68% w/w), wax esters (25%), triacyl glycerol (5%), O-acyl-ω-hydroxy fatty acids (OAHFA) (4%), which are substantially more abundant than adione (0.3 fmol/nmol total cholesterol), ADT (0.05 fmol/nmol) and epiADT (0.03 fmol/nmol) (Figure 4C). Moreover, steroids are structurally similar compounds and also suffer from poor ionization efficiency in MS/MS analysis; thus targeted MS/MS approach with improved sensitivity is required. As such, in this study, we carried out radioactive tracer experiments in combination with pharmacological enzyme inhibition and thereby selected the potential Te metabolites for targeted LC-MS/MS analysis. Moreover, in order to detect trace concentrations of endogenous steroid hormone metabolites, we enhanced the MS/MS sensitivity by introducing an ionizable moiety (QAO) to the ketone group of each target metabolites. Employing the methodologies described above enabled detection of natural steroid hormone metabolites contained in human meibum.

A potential clinical application of our findings may include assessment of local androgen activity in the meibomian gland by quantifying androgen metabolites in meibum. Previous studies demonstrate that the human meibomian gland function is strongly influenced by sex steroid hormones, particularly androgens (see study by Bron et al.^15^ and references therein). In both females and males, androgens promote the synthesis and secretion of meibum lipids and suppress the expression of genes related to keratinization. Conversely, androgen deficiency—such as that seen in aging, Sjögren’s syndrome, antiandrogen treatment, or complete androgen insensitivity syndrome—is associated with meibomian gland dysfunction, altered meibum lipid profiles, and decreased tear film stability.^17^^,^^25^^,^^52^^,^^53^ Accumulating data thus suggest the potential clinical importance of measuring local androgen activity to understand the etiology of meibomian gland dysfunction and to develop therapies for ameliorating this condition.^3^^,^^16^ In this context, we identified specific androgen metabolites in meibum. Meibum is clinically accessible lipid excreta from the meibomian gland. These metabolite markers may help to provide a unique opportunity to assessing local-tissue androgen activity. Although further studies are required, these metabolites may contribute as a clinical surrogate endpoint for assessing meibomian gland dysfunction or dry eye, particularly in the context of drug discovery and development for these unmet medical conditions.^54^^,^^55^

Collectively, in the present study, we have elucidated the molecular identity of the tissue-endogenous androgen metabolism in the meibomian gland. The deactivation of active steroid hormones is equally important as their *de novo* synthesis in determining the time and space of their actions. Our studies contribute to understanding the intracrine system of the meibomian gland, encompassing both the generation and deactivation of local steroid hormones. Our methods used in this study and results/findings may pave the way to understand the local steroid-hormone system reported in other intracrine tissues.

### Limitations of the study

The potential of the identified metabolites, adione, ADT, and epiADT, for assessing local androgen activity in patients with different meibomian gland disorders or symptoms remains to be explored. Our currently available data are only confined to healthy subjects with no eye-related diseases. Although these metabolites have the potential to serve as a clinical surrogate endpoint in the evaluation of meibomian gland dysfunction or dry eye conditions, further research is still required.

## Resource availability

### Lead contact

Further information and requests for resources should be directed to and will be fulfilled by the lead contact, Masao Doi (doimasao@pharm.kyoto-u.ac.jp).

### Materials availability

This study did not generate new unique reagents.

### Data and code availability

• All data reported in this paper will be shared by the lead contact upon request.
• This paper does not report original code.
• Any additional information required to reanalyze the data reported in this paper is available from the lead contact upon request.

## Acknowledgments

We thank Ryosuke Ochiai, Tsubasa Ibushi, and Tomoyo Yoshioka in Shimadzu Techno-Research for their expert assistance in LC-MS/MS studies. This work was supported in part by research grants from the 10.13039/501100001700Ministry of Education, Culture, Sports, Science and Technology of Japan (22H04987, 24H02306, 22K09771, and 24K02178), the Cyclic Innovation for Clinical Empowerment of the Japan Agency for Medical Research and Development (JP22pc0101069), 10.13039/501100007263Astellas Foundation for Research on Metabolic Disorders, 10.13039/501100004330SRF, 10.13039/501100008664Ono Medical Research Foundation, and Japan Endocrine Society Grant for Promising Investigator. K.T.N.P. is supported by a Japan Science and Technology Agency SPRING fellowship.

## Author contributions

M.D. conceived the project; M.D. and K.T.N.P. designed the research with T.S. and S.K.; K.T.N.P. performed experiments in collaboration with T.M., Y.H., H.U., M.M., T.N., and E.H; T.S. and S.K. sampled human meibum samples; M.D., K.T.N.P., and T.M. wrote the paper with input from all authors.

## Declaration of interests

M.D., T.S., and S.K. received a research grant from Senju Pharmaceutical Co., Ltd. M.M. and T.N. are employed by Senju Pharmaceutical Co., Ltd.

## STAR★Methods

### Key resources table

**Table:** 

REAGENT or RESOURCE	SOURCE	IDENTIFIER
**Antibodies**		

Rabbit monoclonal anti-AKR1C3/4	Abcam	Cat# ab209899, RRID:AB_2922995
Mouse monoclonal anti-HSD3B1	Abnova	Cat# H00003283-M01, RRID:AB_425493
Rabbit polyclonal anti-AKR1C4	Affinity Biosciences	Cat# DF9190, RRID:AB_2842386
Donkey anti-rabbit IgG, Alexa Fluor^TM^ 594-conjugated	Thermo Fisher Scientific	Cat# A-21207, RRID:AB_141637
Donkey anti-mouse IgG, Alexa Fluor^TM^ 488-conjugated	Cell Signaling	Cat# A-21202, RRID:AB_141607
Goat anti-rabbit IgG, peroxidase labeled polymer conjugated	Agilent	Cat# K4003, RRID:AB_2630375

**Chemicals, peptides, and recombinant proteins**		

Testosterone, [1,2,6,7-^3^H]	Perkin Elmer	Cat# NET370
5α-dihydrotestosterone, [1,2,4,5,6,7-^3^H]	Perkin Elmer	Cat# NET926
Androst-4-ene-3,17-dione, [1β-^3^H]	Perkin Elmer	Cat# NET453
Dutasteride	Cayman Chemical	Cat# 15956
Fadrozole hydrochloride	Sigma Aldrich	Cat# F3806
Testosterone, used in Figure 3	Tokyo Chemical Industry	Cat# T0027
Testosterone, used in Figure 4	FUJIFILM Wako Pure Chemical	Cat# 201-20551
Androstenedione	Tokyo Chemical Industry	Cat# A0845
Epiandrosterone	Tokyo Chemical Industry	Cat# E0374
Androstanedione, used in Figure 3	Matrix Scientific	Cat# 155569
Androstanedione, used in Figure 4	ALB Technology Limited	Cat# ALB-RS-04085
Androsterone, used in Figure 3	Cayman Chemical	Cat# 15872
Androsterone, used in Figure 4	FUJIFILM Wako Pure Chemical	Cat# 015-03971
5α-androstane-3α,17β-diol (3α-diol)	Sigma Aldrich	Cat# A7755
5α-androstane-3β,17β-diol (3β-diol)	Biosynth AG	Cat# FA17909
Testosterone-d3	Supelco	Cat# T2655
Epiandrosterone-d4	Cambridge Isotope Laboratories, Inc.	Cat# DLM-10269
Androstanedione-d4	ALSACHIM	Cat# C2125
Androsterone-d4	Cambridge Isotope Laboratories, Inc.	Cat# DLM-10402

**Critical commercial assays**		

Amplifex^TM^ Keto Reagent	Sciex	Cat# 4465962
Dako REAL EnVision Detection System, Peroxidase/DAB, Rabbit/Mouse, HRP Kit	Agilent	Cat# K5007, RRID:AB_2888627

**Software and algorithms**		

Prism 8	GraphPad Prism	RRID:SCR_002798
LabSolutions	Shimadzu	RRID:SCR_018241
ProFSA Software	Perkin Elmer	Cat# ProFSA625TR

### Experimental model and study participant details

#### Animals

C57BL/6J male and female mice were purchased from Japan SLC and maintained on a 12-h light:12-h dark cycle with *ad libitum* access to food and water as described.^56^ Mice were sacrificed by cervical dislocation for tissue collection at 3–4 months of age. All procedures were conducted in compliance with the Ethical Regulations of Kyoto University and performed under protocols approved by the Animal Care and Experimentation Committee of Kyoto University (Protocol #24-21).

#### Human participants

For this study, young, healthy Japanese participants aged 21–35 years were recruited from the local populations of Kyoto and Osaka, Japan, through a third-party corporation with no conflicts of interest. Participants confirmed the absence of systemic or ocular diseases via a written interview conducted prior to enrollment. Of the participants, 10 males and 10 females with no signs or symptoms of meibomian gland dysfunction (MGD) or dry eye disease (DE) were selected following an eye examination performed using a slit-lamp microscope. Participants self-reported their sex. The study, which involved clinical data collection and meibum sampling, received approval from the Institutional Review Board of Kyoto Prefectural University of Medicine (KPUM) [Protocol #ERB-C-2649]. All participants provided written informed consent and were enrolled in compliance with the principles of the Declaration of Helsinki. Exclusion criteria included anatomical or functional abnormalities of the eyelids, such as tumors; ocular allergies or infections; the use of punctal plugs or a history of surgical punctal occlusion; eye surgery within the past three months; contact lens use; anti-glaucoma treatments; or ongoing use of local or systemic antimicrobial or steroid medications. Meibum sampling and clinical data collection related to the meibomian glands and ocular surface were performed at Kyoto City Hospital (KCH).

#### Human eyelid specimens

Formalin-fixed, paraffin-embedded human eyelid samples obtained from postmortem donors of 41-year-old male and 34-year-old female, who had no history of eye-related diseases, both purchased from Science Care (Phoenix, Arizona, USA).

### Method details

#### Testosterone radioactive tracing

The isolated upper eyelids (tarsal plates), testes or adrenal glands were incubated *in vitro* in pre-aerated Hank’s balanced salt solution (HBSS) containing either 80 nM ^3^H-labelled Te (Testosterone, [1,2,6,7-^3^H]), DHT ([5α-dihydrotestosterone, [1,2,4,5,6,7-^3^H]), or A4 (androst-4-ene-3,17-dione, [1β-^3^H]) (all from PerkinElmer) for 3 h at 37°C with gentle vibration as described previously.^14^^,^^57^ Where indicated, 10 μM dutasteride (Cayman Chemical) or fadrozole hydrochloride (Sigma) was included in medium. After incubation, steroids were extracted into 1 mL ethyl acetate from the tissues, followed by evaporation to dryness under nitrogen at 75°C with 2 μL propylene glycol as a carrier solvent. The residues were dissolved using 43% acetonitrile (ACN) and filtered through a 0.22 μm PVDF membrane. Samples were analyzed using a Waters e2695 high-performance liquid chromatography (HPLC) system coupled with an online flow scintillation analyzer (625TR series, PerkinElmer). Chromatographic separation was performed on a Lichrospher 100 RP-18 column (5 μm, 250 × 4 mm; Kanto Chemicals) with a LiChroCART guard column (5 μm, 4 × 4 mm; Merck) at 40°C. Gradient elution was performed using a mobile phase consisting of water (solvent A) and ACN (solvent B) at the flow rate of 0.7 mL/min and the following program: 43–46% B (0–30 min), 46–50% B (30–35 min), 50–100% B (35–40 min), 100% B (40–45 min), 100–43% B (45–50 min), and post run 10 min. Peak areas were quantified using the ProFSA software (PerkinElmer) and calculated as percentage of the sum of all peaks for each individual sample to compare data across samples. The RI chromatography in Figure 3A was performed using the same HPLC conditions as the LC-MS chromatography. The tissues boiled at 95°C for 5 min were used for negative control.

#### Testosterone tracing by LC-MS/MS

Non-radioactive testosterone was used for LC-MS/MS-based testosterone tracing. The tissues were incubated under the same conditions as those for radioactive tracing except increased concentration of testosterone (1 μM) and elongation of incubation time (5 h) to allow sufficient quantification of all steroid metabolites by LC-MS/MS. The tissues boiled at 95°C for 5 min were used for negative control. Steroids were measured using a Shimadzu Nexera X2 Ultra HPLC system coupled to a triple quadrupole mass spectrometer (LCMS-8040, Shimadzu) with reference to authentic standards for adione (Matrix Scientific), ADT (Cayman Chemical), 3β-diol (Biosynth AG) and 3α-diol (Sigma-Aldrich), A4, Te and epiADT (all from Tokyo Chemical Industry). Chromatographic separation was performed on a Lichrospher 100 RP-18 column (5 μm, 250 × 4 mm; Kanto Chemicals) with a LiChroCART guard column (5 μm, 4 × 4 mm; Merck) at 40°C. Mobile phase consisted of water (A) and ACN (B), both containing 0.1% formic acid. Using a flow rate of 0.5 mL/min, chromatographic separation was achieved with the gradient elution time program as follows: 43–46% B (0–42 min), 46–50% B (42–49 min), 50–100% B (49–56 min), 100% B (56–63 min), 100–43% B (63–70 min), and 43% B (70–84 min). Metabolites were identified by comparing their retention times and the relative intensities of 3 multiple reaction monitoring (MRM) events with those of the authentic standards. The peak area of each metabolite target was analyzed using LabSolutions software (Shimadzu). The parameters for LC-MS/MS analysis in Figures 3 and S1 are shown in Table S2.

#### LC-MS/MS for human meibum profiling

Meibum was collected as described previously^58^ and dissolved in 0.5 mL chloroform. Samples were stored in sealed glass tubes at −80°C before further processing. Quantitation of human meibum steroid was performed at Shimadzu Techno-Research (Kyoto, Japan). Unlabelled and deuterated standards were purchased from FUJIFILM Wako Pure Chemical Corporation (Te, ADT), Tokyo Chemical Industry (epiADT), ALB Technology Limited (adione), and Sigma-Aldrich Co. LLC (cholesterol). Testosterone metabolites were derivatized with a permanently charged quaternary aminooxy (QAO) reagent to enhance their ESI-MS/MS sensitivity.^59^ For steroid quantification, 100 μL of sample was spiked with 50 μL internal standard (IS) mixture containing 2 ng/mL deuterated Te (testosterone-d3, Supelco), epiADT (epiandrosterone-d4, Cambridge Isotope Laboratories, Inc.), adione (androstanedione-d4, ALSACHIM), and ADT (androsterone-d4, Cambridge Isotope Laboratories, Inc.) prior to evaporation to dryness in a centrifugal evaporator (CVE 3100). The residue was reconstituted in 10 μL QAO reagent solution (Supelco-SigmaAldrich, Kyoto, Japan) and 40 μL methanol containing 7%vol acetic acid. After 1 h of heating at 70°C, the samples were dried using a centrifugal evaporator (CVE 3100). Fifty microliters of water/methanol containing 7%vol acetic acid = 4:1 (v/v) solvent mixture was added and the samples were analyzed on a Shimadzu Nexera X2 Ultra HPLC system coupled to a triple quadrupole mass spectrometer (LCMS-8060, Shimadzu). For the quantification of total cholesterol, 5 μL samples were spiked with 20 μL IS solution containing 25 μg/mL cholesterol-d7, 20 μL methanol and 175 μL KOH (0.35N). After heating at 50°C for 1 h, the samples were neutralized with 200 μL methanol containing 5% acetic acid and filtered. The samples were analyzed on a UPLC-MS-MS system consisting of a Shimadzu Nexera X2 Ultra HPLC system (Shimadzu, Kyoto, Japan) and a 5500QTRAP mass spectrometer (Sciex, Framingham, MA, USA).

Testosterone metabolites derivatized with QAO reagent were analyzed using a Shim-pack Scepter C18-120 column (1.9 μm, 2.1 mm I.D. × 100 mm; Shimadzu, Kyoto, Japan). Mobile phase A was ultrapure water containing 0.1%vol formic acid and 2 mmol/L ammonium formate; mobile phase B was acetonitrile/water = 95/5 containing 0.1%vol formic acid and 2 mmol/L ammonium formate. The flow rate was set to 0.35 mL/min and the HPLC gradient was as follows: 0–0.75 min, 25% B; 0.75–13.0 min, 25–40% B; 13.0–14.0 min, 40–90% B; 14.0–17.0 min, 90% B; 17.0–17.1 min, 90-25% B; 17.1–20.0 min, 25% B. The MRM transitions and retention times for all compounds analyzed are shown in Table S3.

Cholesterol was analyzed using a TSKgel column (5 μm, 2.0 mm I.D. × 50 mm; Tosoh, Japan). Mobile phase A was ultrapure water containing 0.1%vol formic acid; mobile phase B was acetonitrile containing 0.1%vol formic acid. The flow rate was set at 0.5 mL/min, and the HPLC gradient was as follows: 0.0–3.0 min, 90–100% B; 3.0–6.0 min, 100% B; 6.0–6.1 min, 100-90% B; 6.1–8.0 min, 90% B. The MRM transitions and retention times for all compounds analyzed are shown in Table S3.

The linearity of the method was determined by analysis of standard plots associated with a freshly prepared seven-point standard calibration curve. Stock solutions of standards and internal standards were prepared in chloroform/methanol [2:1, (v/v)]. Calibration standard (CS) samples were prepared to give concentrations: 1.5, 7.5, 15, 75, 150, 750 and 1500 pg/mL for steroids and 4, 20, 40, 200, 400, 2000 and 4000 μg/mL for cholesterol, while quality control (QC) samples were prepared with rabbit meibum at three concentrations: 15, 150 and 750 pg/mL for steroids and 40, 200 and 400 μg/mL for cholesterol. The peak area ratios of analyte/IS compared to the nominal concentrations of each calibration standard point were plotted using a linear regression with a weighted factor of 1/x^2^ to calculate the concentration of each compound in the samples. The linear regression equation, correlation coefficient (*r*), linear range, and LOD are presented in Table S4.

#### Immunohistochemistry and immunofluorescence staining

Five-μm-thick eyelid paraffin sections were antigen-retrieved by pressure cooking in Tris-EDTA buffer (pH 9.0) as described^14^ and immersed into PBS containing 0.1% Tween 20 (PBS-T). Sections were blocked with 3% BSA in PBS-T for 2 h and incubated with a specific set of antibodies, including anti-AKR1C3+AKR1C4 antibody (rabbit monoclonal, EPR16726, Abcam, final concentration 0.4 μg/mL for IHC and 0.7 μg/mL for IF) and anti-HSD3B1 (mouse monoclonal, 3C11-D4, Abnova, final 0.05 μg/mL for IHC and 0.5 μg/mL for IF)^60^ for 24 h at 4°C. The immunoreactivities were visualized with 3,3-diaminobenzidine using horseradish peroxidase-labeled anti-IgG polymers (Dako, EnVision+ System-HRP Labeled Polymer anti-rabbit for AKR1C3+AKR1C4 and anti-mouse for HSD3B1) or visualized using Alexa 594-conjugated anti-rabbit (for AKR1C3+AKR1C4) or anti-mouse (for HSD3B1) IgG (Thermo Fisher Scientific, 1:1000 dilution).^61^ For immunofluorescence, sections were mounted in medium containing 4ʹ,6-diamidino-2-phenylindole (DAPI) for counterstaining cell nuclei.

### Quantification and statistical analysis

Statistical analysis was performed using GraphPad Prism 8. two-way ANOVA followed by Bonferroni’s post-hoc tests were used to analyze statistical significance between groups. The results and details of statistics are available in corresponding figure legends and Table S5. The results that reach statistical significance are indicated by ∗ or # in the figures.
