# Supplementary material for: Identification of meibomian gland testosterone metabolites produced by tissue-intrinsic intracrine deactivation activity
Source: iScience. 2025 Jan 27;28(2):111808. doi: 10.1016/j.isci.2025.111808 (PMC11848505; doi:10.1016/j.isci.2025.111808)
Supplement: Document S1. Figures S1–S3 and Tables S1–S5 [file mmc1.pdf]

iScience, Volume *28*

Supplemental information

Identification of meibomian gland testosterone metabolites produced by tissue-intrinsic intracrine deactivation activity

Khanh Tien Nguyen Pham, Takahito Miyake, Tomo Suzuki, Shigeru Kinoshita, Yuki Hamada, Hikari Uehara, Mamiko Machida, Takeshi Nakajima, Emi Hasegawa, and Masao Doi

A Androstenedione (A4) (peak a)

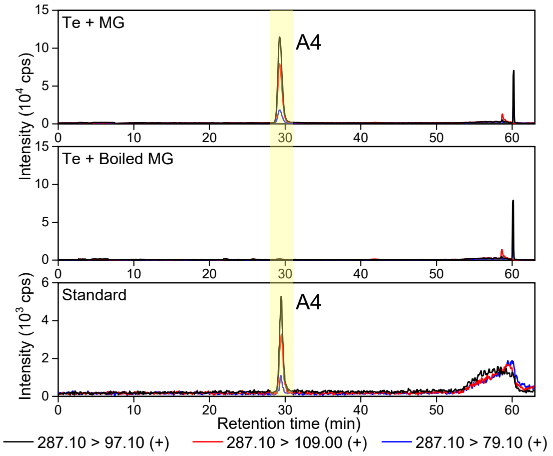

MRM transition	Standard relative intensity	Te metabolites relative intensity
287.10 > 97.10	1.00	1.00
287.10 > 109.00	0.61	0.66
287.10 > 79.10	0.15	0.15

B Epiandrosterone (epiADT) (peak X)

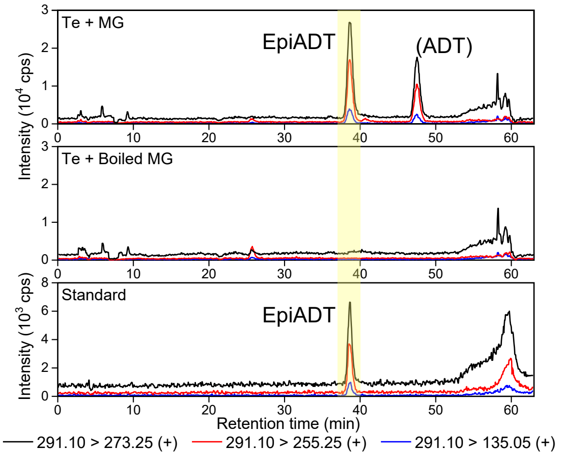

MRM transition	Standard relative intensity	Te metabolites relative intensity
291.10 > 273.25	1.00	1.00
291.10 > 255.25	0.50	0.57
291.10 > 135.05	0.13	0.08

C Androstanedione (adione) (peak Y)

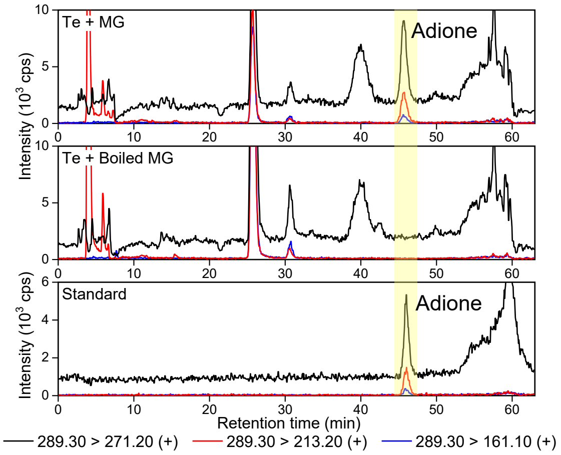

MRM transition	Standard relative intensity	Te metabolites relative intensity
289.30 > 271.20	1.00	1.00
289.30 > 213.20	0.11	0.24
289.30 > 161.10	0.04	0.04

D Androsterone (ADT) (peak Z)

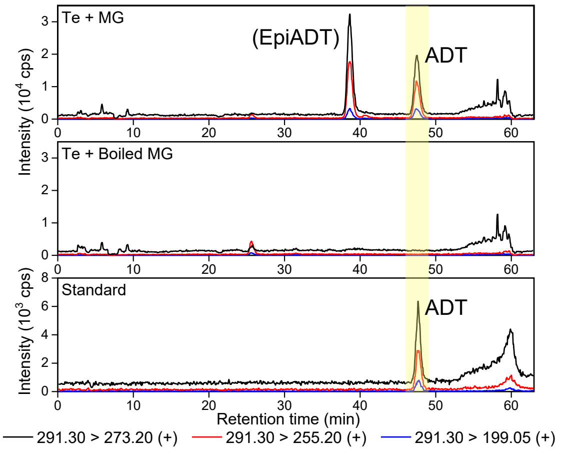

MRM transition	Standard relative intensity	Te metabolites relative intensity
291.30 > 273.20	1.00	1.00
291.30 > 255.20	0.54	0.61
291.30 > 199.05	0.11	0.09

Figure S1. Chromatograms of 3 MRM transitions for androstenedione, epiandrosterone, androstanedione, and androsterone, related to Fig. 3. The upper panels represent LC-ESI-MS/MS analysis of extraction from the meibomian gland after incubation with Te. The middle panels represent LC-ESI-MS/MS analysis of the negative control extraction from boiled meibomian gland after incubation with Te. The lower panels represent LC-ESI-MS/MS analysis of authentic standards.
(A) MRM transitions for detecting A4 were 287.10 > 97.10 (black), 287.10 > 109.00 (red), and 287.10 > 79.10 (blue).
(B) MRM transitions for detecting epiADT were 291.10 > 273.25 (black), 291.10 > 255.25 (red), and 291.10 > 135.05 (blue).
(C) MRM transitions for detecting adione were 289.30 > 271.20 (black), 289.30 > 213.20 (red), and 289.30 > 161.10 (blue).
(D) MRM transitions for detecting ADT were 291.30 > 273.20 (black), 291.30 > 255.20 (red), and 291.30 > 199.05 (blue).
Metabolites were identified by comparing the relative intensities of 3 independent multiple reaction monitoring (MRM) events with those of the standards.

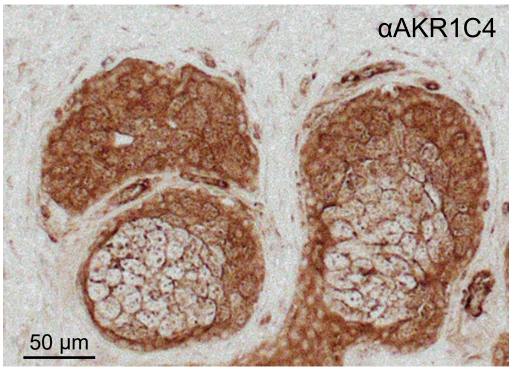

Figure S2. Immunohistochemistry for anti-AKR1C4, related to Fig. 4. A polyclonal rabbit antibody, raised against human AKR1C4 (Affinity Biosciences, #DF9190), was used. Specimen, 41-year-old male eyelid section.

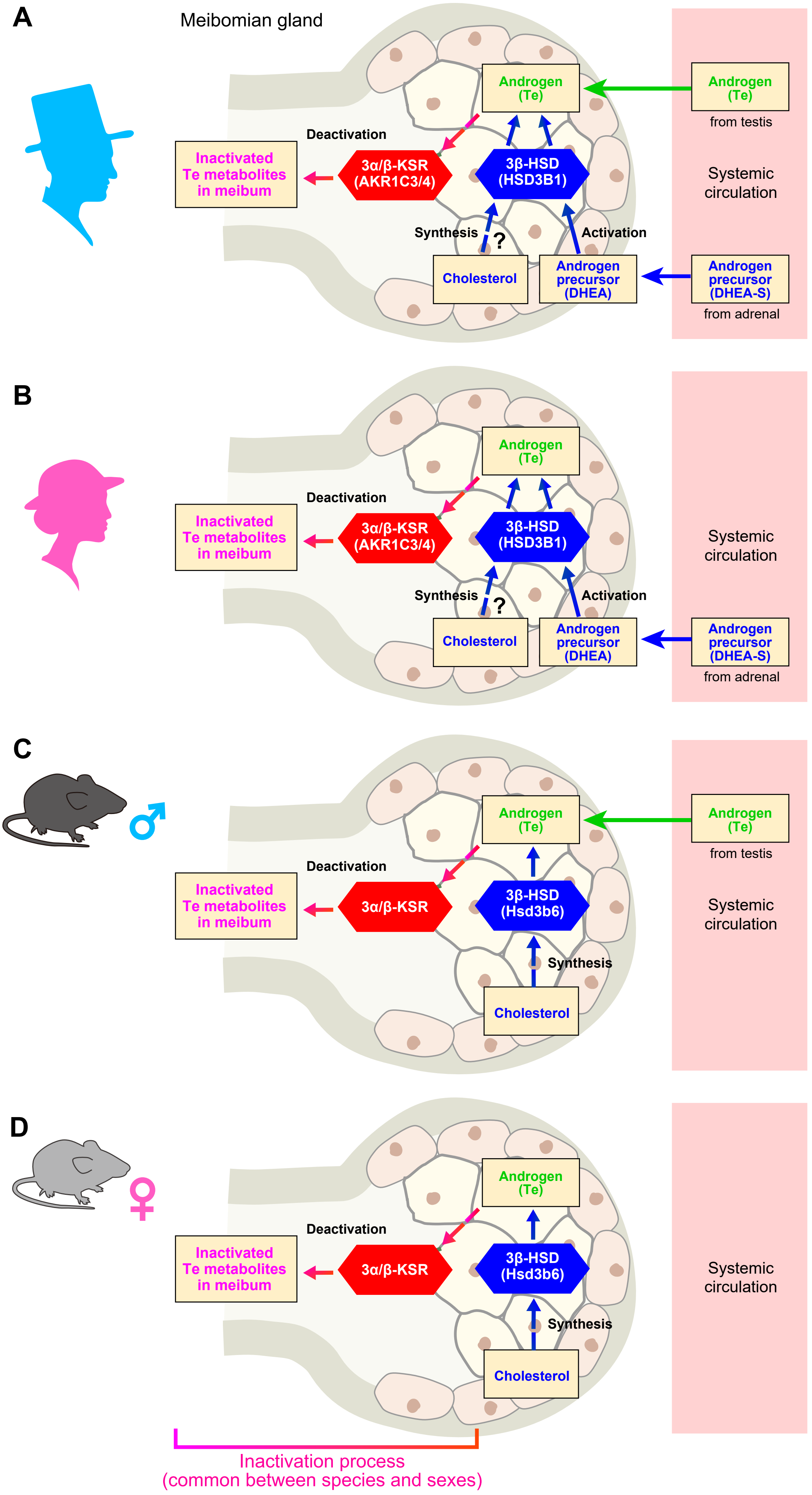

Figure S3. Schematics showing meibomian gland intracrinology, related to Figs. 1–4. Schematic model showing androgen (Te) inactivation process in the meibomian gland in human male (A), female (B), mouse male (C), and female (D). The process of deactivation is common between species and sexes. On the other hand, androgen supplying process exhibits notable differences between human and mouse and between male and female, as reported in the literature[2,3,15].

Table S1

Data table of Te metabolites in human meibum specimens shown in Fig. 4C

Gender	Age	Sample name[#]	Relative abundance (fmol/nmol total cholesterol)				Cholesterol level (nmol)
			Te	Adione	ADT	EpiADT	
Male	27	N005	0.0132*	0.2693	0.0428	0.0304	300.0
	21	N006	0.0013*	0.3030	0.0696	0.0236	307.8
	25	N007	0.0031*	0.2805	0.0539	0.0199	336.2
	27	N009	0.0000*	0.4060	0.0611	0.0262	452.6
	22	N012	0.0102*	0.3887	0.0650	0.0358	359.5
	33	N014	0.0100	0.2843	0.0405	0.0229	600.0
	35	N015	0.0135	0.4253	0.0488	0.0296	419.0
	34	N016	0.0123*	0.3280	0.0570	0.0308	434.5
	33	N023	0.0224*	0.2899	0.0714	0.0446	226.0
	30	N036	N/A	0.2619	0.0130*	N/A	61.0
Female	26	N001	0.0031*	0.1389	0.0204	0.0127*	356.9
	25	N002	0.0063*	0.4285	0.0953	0.0330	315.5
	25	N003	0.0000*	0.2681	0.0403	0.0328	369.8
	28	N004	0.0021*	0.1288	0.0187*	0.0168*	218.0
	30	N010	0.0000*	0.3767	0.0977	0.0223*	171.2
	24	N011	0.0000*	0.4087	0.0618	0.0215	747.4
	23	N013	0.0119*	0.2295	0.0710	0.0377	305.2
	34	N018	0.0330	0.1129	0.0057*	0.0196*	214.9
	35	N019	0.0033*	0.2643	0.0458	0.0154	1262.1
	22	N020	0.0150*	0.3411	0.0489	0.0255	261.2

*, lower than limit of quantification (< LOQ); N/A was due to a small size of this sample material (c.f. cholesterol amount); #Sample names are temporarily assigned for specimen identification purposes in the data presentation of this study.

Table S2

Mass spectrometric parameters for Te metabolites in mouse meibomian glands, related to Fig. 3

Analyte	Precursor ion (m/z)	Product ion (m/z)	Collision Energy (eV)	Retention time (min)
Testosterone (Te)	289.30	97.00	25.0	25.8
		109.00	25.0	
		79.00	46.0	
Androstenedione (A4)	287.10	97.10	23.0	29.3
		109.00	22.0	
		79.10	49.0	
Epiandrosterone (epiADT)	291.10	273.25	9.0	38.8
		255.25	14.0	
		135.05	21.0	
Androstanedione (adione)	289.30	271.20	11.0	45.9
		213.20	15.0	
		161.10	30.0	
Androsterone (ADT)	291.30	273.20	10.0	47.6
		255.20	16.0	
		199.05	21.0	
5α-androstane-3α,17β-diol (3α-diol)	257.10	161.10	15.0	37.0
		81.05	31.0	
		147.15	18.0	
5α-androstane-3β,17β-diol (3β-diol)	257.30	81.10	30.0	32.2
		147.00	19.0	
		161.15	19.0	

Table S3

Mass spectrometric parameters for Te metabolites in human meibum specimens, related to Fig. 4

Analyte	Precursor ion (m/z)	Product ion (m/z)	Collision Energy (eV)	Retention time (min)
QAO-Te	403.3	164.1	44	8.1 and 9.1
QAO-Te-d3	406.3	164.1	44	8.0 and 9.0
QAO-epiADT	405.3	161.1	38	8.4
QAO-epiADT-d4	409.3	161.1	38	8.3
QAO-adione	259.4	288.3	23	4.6 and 5.0
QAO-adione-d4	261.4	292.1	24	4.6 and 5.0
QAO-ADT	405.3	161.1	38	11.5
QAO-ADT-d4	409.3	161.1	38	11.4
Cholesterol	369	161	22	2.6
Cholesterol-d7	376	161	22	2.6

Table S4

Mass spectrometric quantification for Te metabolites in human meibum specimens, related to Fig. 4

Analyte	Linear regression equation	Correlation coefficient (*r*)	Linear range (pg/ml)*	LOD (pg/ml)
Te	y = 0.00107 x + 0.00175	0.9998	1.50 – 1500	0.00935
EpiADT	y = 0.000796 x + 0.00319	0.9987	1.50 – 1500	0.0126
Adione	y = 0.00129 x + 0.00149	0.9998	1.50 – 1500	0.00775
ADT	y = 0.000821 x + 0.00202	0.9994	1.50 – 1500	0.0122
Cholesterol	y = 0.00533 x + 0.00193	0.9988	4 – 4000 (ng/ml)	N/D

LOD, limit of detection; N/D: Not determined. *Cholesterol was measured in ng/ml.

Table S5

Statistical information, related to Figs. 2 and 4

Figure	Test	Information (F, *P* value, and number of biological units)
Fig. 2B (DMSO vs Dutasteride)	Two-way ANOVA	Factor 1 (metabolites) $F(3, 32) = 10.36$, $P < 0.0001$; Factor2 (inhibitor) $F(1, 32) = 0.0006990$, $P = 0.9791$; Interaction $F(3, 32) = 25.33$, $P < 0.0001$; $n = 5$
	Bonferroni's multiple comparisons test	metabolite a, $P < 0.0001$; metabolite X, $P = 0.5378$; metabolite Y, $P = 0.0023$; metabolite Z, $P = 0.2135$; $n = 5$
Fig. 2B (DMSO vs Fadrozole)	Two-way ANOVA	Factor 1 (metabolites) $F(3, 32) = 72.17$, $P < 0.0001$; Factor2 (inhibitor) $F(1, 32) = 1.242$, $P = 0.2734$; Interaction $F(3, 32) = 1.268$, $P = 0.3020$; $n = 5$
	Bonferroni's multiple comparisons test	metabolite a, $P > 0.9999$; metabolite X, $P > 0.9999$; metabolite Y, $P = 0.1534$; metabolite Z, $P > 0.9999$; $n = 5$
Fig. 2C (DMSO vs Dutasteride)	Two-way ANOVA	Factor 1 (metabolites) $F(3, 16) = 10.88$, $P = 0.0004$; Factor2 (inhibitor) $F(1, 16) = 0.3301$, $P = 0.5736$; Interaction $F(3, 16) = 8.068$, $P = 0.0017$; $n = 3$
	Bonferroni's multiple comparisons test	metabolite a', $P = 0.007$; metabolite X', $P > 0.9999$; metabolite Y', $P = 0.0437$; metabolite Z', $P = 0.896$; $n = 3$
Fig. 2C (DMSO vs Fadrozole)	Two-way ANOVA	Factor 1 (metabolites) $F(3, 16) = 9.763$, $P = 0.0007$; Factor2 (inhibitor) $F(1, 16) = 0.01555$, $P = 0.9023$; Interaction $F(3, 16) = 0.8766$, $P = 0.4738$; $n = 3$
	Bonferroni's multiple comparisons test	metabolite a', $P = 0.6513$; metabolite X', $P > 0.9999$; metabolite Y', $P > 0.9999$; metabolite Z', $P > 0.9999$; $n = 3$
Fig. 2D	Two-way ANOVA	Factor 1 (metabolites) $F(3, 16) = 13.65$, $P = 0.0001$; Factor2 (inhibitor) $F(1, 16) = 0.008659$, $P = 0.9270$; Interaction $F(3, 16) = 0.2079$, $P = 0.8894$; $n = 3$
	Bonferroni's multiple comparisons test	metabolite DHT, $P > 0.9999$; metabolite X'', $P > 0.9999$; metabolite Y'', $P > 0.9999$; metabolite Z'', $P > 0.9999$; $n = 3$
Fig. 4C	Two-way ANOVA	Factor 1 (metabolites) $F(3, 72) = 153.4$, $P < 0.0001$; Factor2 (gender) $F(1, 72) = 1.846$, $P = 0.1785$; Interaction $F(3, 72) = 1.419$, $P = 0.2443$; $n = 10$
	Bonferroni's multiple comparisons test	metabolite Te, $P > 0.9999$; metabolite Adione, $P = 0.0642$; metabolite ADT, $P > 0.9999$; metabolite epiADT, $P > 0.9999$; $n = 10$
